# *In situ* X-ray area detector flat-field correction at an operating photon energy without flat illumination

**DOI:** 10.1107/S1600577523001157

**Published:** 2023-03-10

**Authors:** James Weng, Wenqian Xu, Kamila M. Wiaderek, Olaf J. Borkiewicz, Jahui Chen, Robert B. Von Dreele, Leighanne C. Gallington, Uta Ruett

**Affiliations:** a X-ray Science Division, Advanced Photon Source, Argonne National Laboratory, Lemont, IL 60439, USA; Paul Scherrer Institut, Switzerland

**Keywords:** flat-field correction, flat-field calibration, dectector drift

## Abstract

Flat-field calibration of X-ray area detectors is a challenge due to the inability to generate an X-ray flat-field at the selected photon energy the beamline operates at, which has a strong influence on the measurement behavior of the detector. A method is presented in which a simulated flat-field correction is calculated without flat-field measurements. Instead, a series of quick scattering measurements from an amorphous scatterer is used to calculate a flat-field response. The ability to rapidly obtain a flat-field response allows for recalibration of an X-ray detector as needed without significant expenditure of either time or effort. Area detectors on the beamlines used, such as the Pilatus 2M CdTe, PE XRD1621 and Varex XRD 4343CT, were found to have detector responses that drift slightly over timescales of several weeks or after exposure to high photon flux, suggesting the need to more frequently recalibrate with a new flat-field correction map.

## Abstract 2

A method for calculating the flat-field response of an X-ray area detector using a series of diffraction measurements from an amorphous scatterer, allowing rapid recalibration of an area detector at any selected X-ray energy, is presented.

## 1. Introduction

### 1.1. Motivation

Flat-field calibration of X-ray area detectors at beamlines is a challenge due to the inability to generate an X-ray flat-field at a selected photon energy. Detector flat-field responses are thus often collected using flat-fields generated by X-ray fluorescence and then extrapolated to the photon energy used at the beamline (Skinner *et al.*, 2012 ▸; Moy *et al.*, 1996 ▸; Veale *et al.*, 2013 ▸; Midgley & Schleich, 2015 ▸). Detectors that have been in service for extended periods of time at beamlines often show radiation damage; pixels heavily exposed to high photon flux have significantly different responses compared with the surrounding pixels and show up as a burnt-in image on the detector. In severe cases, these after-images may be clearly visible in dark measurements on the detector. Pixels that have been obscured for long periods of time (*e.g.* by the beamstop) have significantly different responses from exposed pixels when used again after a change in the setup, and will display an artifact such as an apparent shadowing on the detector. In medical imaging using similar detectors, it is known that simply translating the detector results in erroneous apparent shadowing of an imaged object (Park & Sharp, 2015 ▸), also resulting in the need for flat-field corrections (FFCs). The limitations of these detectors when used in medical and industrial imaging are well known, with numerous approaches taken to correct for them (Park *et al.*, 2014 ▸; Cao & Peter, 2008 ▸; Hofmann *et al.*, 2011 ▸). Like the shadowing artifacts in medical imaging with area detectors, similar artifacts are present for diffraction measurements using an area detector; simple translation of an area detector yields significant variations of an integrated 1D pattern for powder diffraction, resulting in the observation of either false peak shifts or a detector-position-dependent phase change. For techniques that rely on subtle changes in baseline shifts to detect changes in structure such as pair distribution functions (PDFs), biological small-angle scattering and contrast variation analysis, faulty measurements caused by improper detector flat-field calibration almost certainly lead to inaccurate subsequent analysis. In this work, detector responses have also been found to drift by a measurable amount on the time scale of several weeks, potentially accumulating in such a way as to cause erroneous measurements.

Here we present a method to rapidly measure and calculate the flat-field response for an X-ray area detector at the selected photon energy at which a given beamline operates. This method, along with the code provided, is not detector-specific, and has no special sample or environment requirements for the measurement procedure. The only requirements for calculating an FFC map in the present work are the ability to translate the detector perpendicular to the incident beam, a calibration standard and an amorphous scatterer. For the amorphous scatterer, common glass microscope slides are found to be sufficient for the purposes of collecting an FFC map.

## 2. Calculation approach

### 2.1. Problem statement

The idealized 2D diffraction pattern measurement without detector errors, *S*(*x*, *y*), can be described as the product of the measured diffraction intensity *I*_m_(*x*, *y*) and the detector flat-field response *G*(*x*, *y*) plus an error term ɛ [equation (1)],

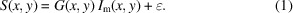

The assumption is made that the detector flat-field response *G* is constant over the time scales relevant to a diffraction measurement; a failure of this assumption implies that the detector cannot be used for the measurements. It is also assumed that the detector response is linear, which means the count from an X-ray counter is linearly proportional to the X-ray dose it receives; if the detector response is far from linear it is likely to be unsuitable for experimental measurements in general. For the detectors used on the beamlines at Sector 11 at the Advanced Photon Source (APS), the Perkin Elmer XRD 1621 and Varex XRD 4343CT are specified to be linear within 1% over their full scale range (PerkinElmer, 2008 ▸; Varex Industrial, 2021 ▸). The Pilatus 3 X 2M CdTe has a factory software correction applied and is also specified to be linear within 1% (Dectris, 2016 ▸).

If *S* is known, without considering the measurement error ɛ, then the FFC map of the detector is given by

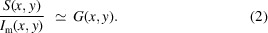

The errors in detector measurement ɛ are normally distributed and average to zero, so they can be ignored if enough repeat measurements are taken. Note that ɛ here is defined to be the random error present in every measurement, and does not include systematic errors such as a radiation-damaged detector that always reads high. In order to obtain an FFC map, a signal *S* with known scattering properties must be measured. Ideally, for simplicity *S*(*x*, *y*) should be a uniform flat-field, making the measured signal simply the flat-field response according to equation (2). However, it is not necessarily possible to generate a uniform flat-field at the selected X-ray energy. In this situation, a sample with known scattering properties that results in a signal *S* with known properties must be used. Knowledge about the sample structure is not necessary. The signal *S*(*x*, *y*) generated must fulfill several criteria to be useful for the calculation of an FFC map using equation (2). It must not be sparse over *x* and *y*, it must be primarily made up of non-zero values over *x* and *y*, or *G*(*x*, *y*) is indeterminate at zero values according to equation (2). *S* must also be a value which the detector can accurately capture; a signal with large jumps in intensity from the baseline may result in a value which is not measurable within the dynamic range of the detector. The attenuation of the scattered beam by the sample has to be constant for the same angles, which is true for spheres and flat objects, but not for capillaries. An amorphous scatterer that produces smooth and very broad diffraction rings (*e.g.* a glass slide) fulfills these requirements.

### 2.2. Description of the algorithm

**Figure 1:**
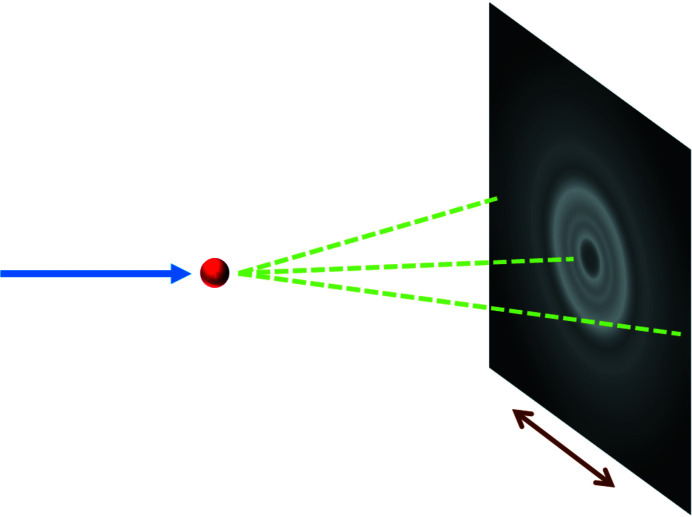
Illustration of the scattering experiment used to collect data needed for an FFC calculation. The incident X-ray beam originates from the left and is shown as a blue arrow. An amorphous scatterer (red sphere) such as glass scatters X-rays (green dashes), resulting in a radially symmetric scattering pattern on the detector, shown on the right. The detector is translated in directions perpendicular to the incident X-ray beam (brown arrow) and multiple measurements are taken.

For the purposes of this description, 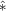 denotes a computed estimate of 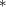 . A measurement is taken of an amorphous scatterer, assumed to have a radially constant scattering pattern under an unpolarized incident beam, with the detector translated to several positions changing the center of the diffraction rings (Fig. 1 ▸). At each of these positions a calibration powder standard is measured to accurately determine the center and detector-to-sample distance to convert the *x*, *y* pixel positions to 2θ positions.

**Figure 2:**
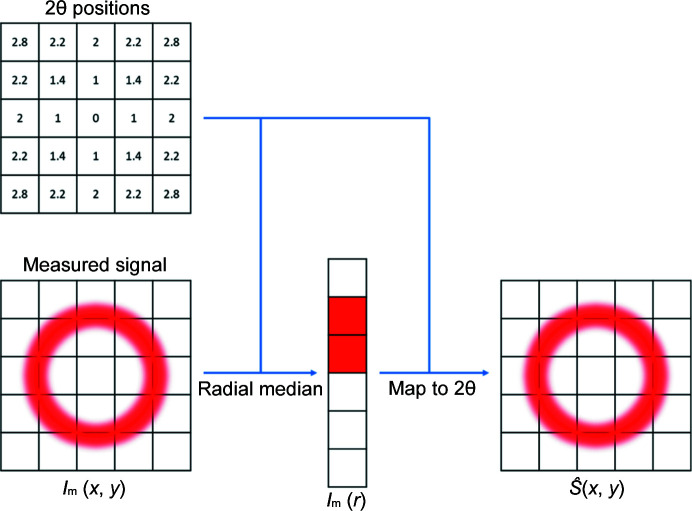
Process by which the estimated idealized 2D scattering signal is calculated by reduction of a radially symmetric 2D scattering pattern to a 1D pattern by taking a radial median and mapping it to computed 2θ positions for each pixel in the original 2D image. An example of a pixel to 2θ position map obtained from a calibration is shown in the top left which is used to compute the radial median.

Using this map, from the measured signal *I*_m_(*x*, *y*) after correction of the beam polarization, a radial average *I*_m_(*r*) of the amorphous scatterer is calculated to provide a 1D scattering pattern. Here, a median is used as the representative radial average over the distance to the center, as outliers are expected if individual pixels read much higher or lower than the rest of the detector prior to FFC. A comparison of the radial mean with the median is shown in Fig. S4 of the supporting information. A truncated mean may also work in this case, though this would require more careful inspection of the detector before calibration to determine the appropriate truncation. A radial median is used by default as a significant number of outliers are frequently observed on detectors which have sustained radiation damage. *I*_m_(*r*) is then mapped back to create an estimate of the signal 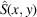 . From equation (2), this allows a first approximation of *G*(*x*, *y*), *i.e.* 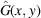 , to be calculated. This process is outlined as a flow chart in Fig. 2 ▸.

**Figure 3:**
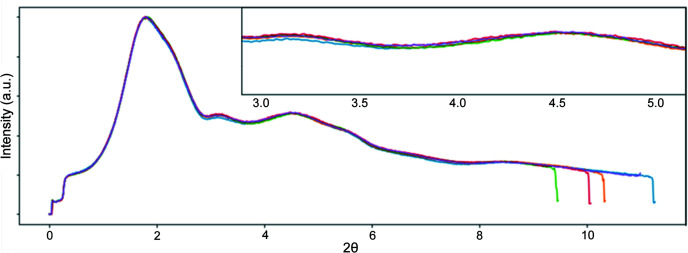
Multiple radial averages of some amorphous scatterer with the detector translated slightly between each measurement showing apparent changes in relative scattering intensity when using an outdated FFC. Changes in relative intensities can lead to incorrect conclusions about the structure, particularly in systems sensitive to low spatial frequency changes in the measured pattern such as PDF measurements on liquids. A Pilatus 2M CdTe detector was used. Intensities on the vertical axes are arbitrary, so no numbers are shown. Vertical magnification of the inset is 2×.

The radial averages of the same amorphous scatterer with the detector translated vary slightly due to an incorrect flat-field response, resulting in an apparent slight shift in the scattering profiles of the measured 1D pattern. For example, with powder X-ray measurements the region surrounding the usual beamstop position in the center is exposed to higher photon flux than the rest of the detector and sustains more damage over time, resulting in a larger change in the flat-field response at those pixels relative to the rest of the detector. For any experimental setup frequently used to measure similar materials, different regions on the detector may consistently receive a higher photon flux. Without FFC, it is expected that multiple radial averages with the detector translated will have shifted peaks and erroneous intensities. This is easily observed by plotting multiple radial averages of the same scatterer where the only difference is detector translation (Fig. 3 ▸), and where peak intensity changes are sufficient to create apparent isosbestic points in the data that do not correspond to any real changes in the scatterer.

**Figure 4:**
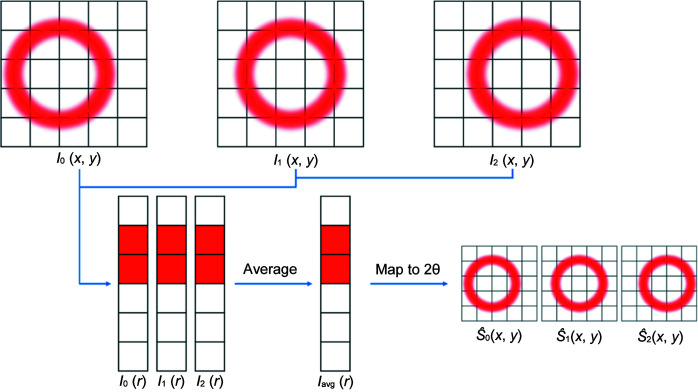
Estimation of an idealized 2D scattering signal by taking the average of multiple radial medians with the detector translated to several positions. The computation with several detector positions is needed to account for any systematic radially dependent changes in the detector flat-field response caused by repeated measurements with the detector at the same position.

In order to correct for this in the final estimate of 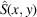 , the amorphous scatterer is measured with the detector translated to multiple positions normal to the incident beam to measure multiple *I*_m_ values. This procedure is outlined in Fig. 4 ▸.

**Figure 5:**
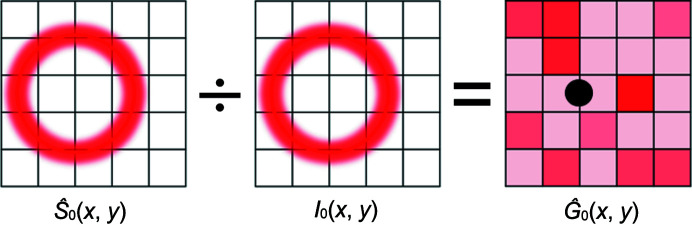
Division of an idealized scattering pattern, 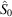 , for a given position 0 by an experimentally measured scattering pattern, *I*_0_, at the same position produces a position-specific FFC map 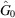 . The position-specific FFC map has no values over any regions obscured by the beamstop, denoted by a black circle. Any asymmetric scattering in the experimental setup will appear in 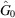 as an erroneous apparent response that does not correspond to an actual detector flat-field response.

The 1D patterns *I*_m_(*r*) for each translated detector position are averaged to obtain a better estimate of *S*. Using the previously calculated *x*, *y* → 2θ map, the averaged *I*_avg_(*r*) is mapped back to 2D for each measured position, providing an estimated 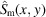 for each scattering measurement. Using equation (2), an estimated *G*_m_(*x*, *y*) is calculated at each position *m*, which contains both null values where the detector is obscured by the beamstop, as well as a ring of erroneous values surrounding the beamstop caused by asymmetric scattering of, partial obstruction of the scattering profile by, or some other unknown artifact created by the beamstop. Both these artifacts were also observed in the literature using similar approaches to measure X-ray detector FFCs (Wernecke *et al.*, 2014 ▸). This process of calculating a position-specific FFC map 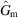 is shown in Fig. 5 ▸.

In order to remove these artifacts, the median of all 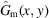 is computed to provide an estimated 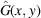 which contains neither null values nor scattering artifacts which originate from the asymmetrical scattering from the beamstop. The presence of such artifacts can additionally be visualized by viewing the absolute difference between a position-specific FFC map and the median FFC map of the detector,

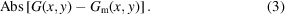

The absolute difference for any given 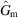 from 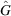 produces an image showing circular artifacts which are concentric to the beamstop position. Inclusion of the information contained at these points would lead to an erroneous computed FFC map 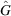 .

Multiple position-specific FFC maps ( 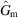 ) with the artifacts removed are then combined to produce an FFC map, 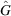 , containing neither the region occluded by the beamstop nor the asymmetric scattering from the beamstop. This is achieved by simply taking the median of several position-specific FFC maps.

**Figure 6:**
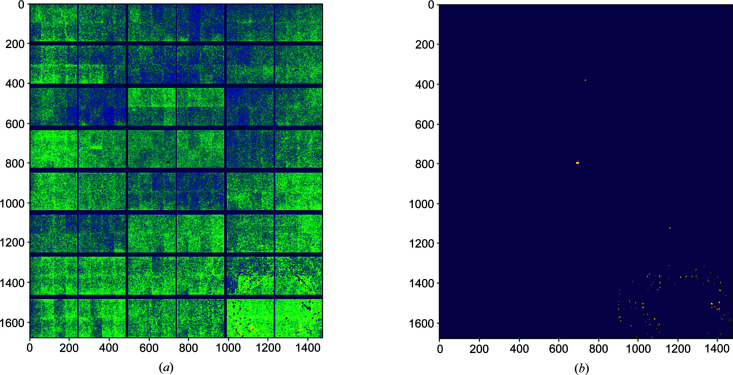
Calculated FFCs for a Pilatus 2M detector with (*a*) normal-responding pixels and (*b*) heavily radiation-damaged pixels separated. Heavily radiation-damaged pixels are defined as those with calculated flat-field responses taken from standard deviations of the average flat-field response of the detector.

From here, damaged pixels are separated from the FFC map. Damaged pixels are those which are identified as having an apparent flat-field response which deviates from the median flat-field response of the detector by more than four standard deviations (σ). These pixels are separated to produce an FFC containing only undamaged pixels and a second map containing only damaged pixels (Fig. 6 ▸).

A median filter is then applied to the FFC map of undamaged pixels in order to reduce the contribution of shot noise. The window size used for the median filter is selected to be no larger than the measured point spread of the detector. In the case of PerkinElmer 1621 or Varex 4343 detectors, the median filter window size used is 7 × 7 pixels, and, for Pilatus 2M CdTe, the median filter window size used is 3 × 3 pixels. The point spread functions of the detectors are measured by illuminating a single pixel with an attenuated X-ray beam cut down to less than 1 pixel in size. For the scintillator-based PerkinElmer 1621 and Varex 4343 detectors, the spread is measured to be a region of at least 10 × 10 pixels, whereas for the Pilatus 2M Cd the spread is measured to be a region of at least 3 × 3 pixels.

A combined FFC map containing corrections of the damaged and undamaged pixels is created by summing the median filtered map of the undamaged pixels with the unmodified map of the damaged pixels. An alternative way of treating the damaged pixels is to exclude them from data analysis, and the map of damaged pixels [*e.g.* Fig. 6 ▸(*b*)] can be used as a mask. It should be determined whether the damaged pixels provide useful measurements with some large apparent correction or erroneous measurements when deciding how to handle them in later data analysis.

### 2.3. Experimental

A series of scattering patterns was collected using an amorphous scatterer with the detector placed quite far from the sample. For measurements taken at 17-BM-B, 11-ID-B and 11-ID-C at the Advanced Photon Source, the sample-to-detector distance used was 1000 mm. The detector distance was chosen such that there was still scattering intensity at the edges of the detector. The amorphous scatterer used in all experiments was a stack of 1.5 mm glass microscope slides. The number of microscope slides used for each measurement was selected so that the scattering intensity across the detector was within the linear regime of the detector. Scattering patterns were taken with the detector at five different positions (Section S1 of the supporting information). Detector translation was arbitrary and was found to be not critical in the calculation of the FFC map, provided the region occluded by the beamstop did not overlap between measurements. A calibration standard was also measured at each position so that a map of pixel to 2θ positions could be calculated using *GSAS-II* (Toby & Von Dreele, 2013 ▸). *GSAS-II* was also used to generate a pixel map of intensity scale due to polarization, so that the polarization-induced intensity difference at different azimuth angles could be corrected. An FFC map was then calculated from these measurements with the software provided by Weng (2022 ▸). It appears that the sample-to-detector position used is not critical to the calculation of an FFC map, provided the scattering intensity across the detector is not attenuated by air scattering to an intensity below the linear response of the detector. Scattering conditions used at various beamlines along with the detectors used are provided in Table 1 ▸. Note that the detector should be allowed to sit between measurements for at least the duration of the measurement in order to minimize burn effects that alter the measurement. Also, beam polarization should be accurately determined so that polarization-induced intensity difference can be correctly removed before FFC map calculation. Beam polarization can be precisely obtained from 2D scattering data of an amorphous material such as a stack of glass slides, as done in this study, using a recently published method (Von Dreele & Xu, 2020 ▸).

## 3. Results

### 3.1. Detector response

**Figure 7:**
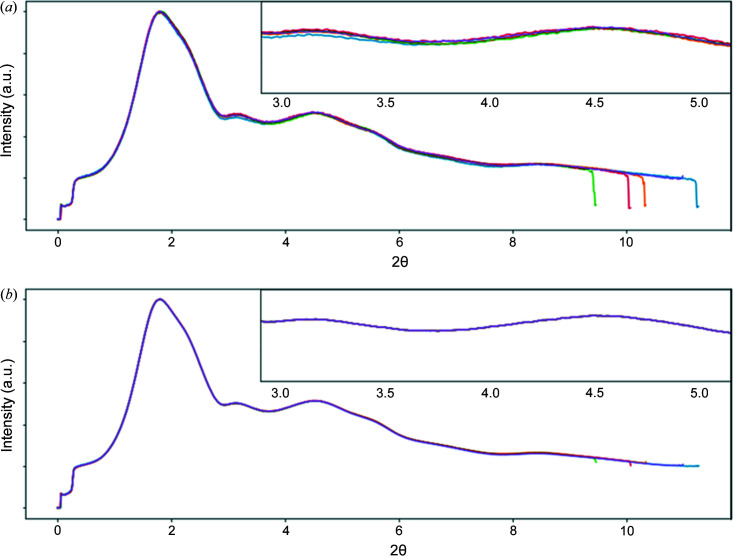
Radial averages of an amorphous scatterer measured using a Pilatus 2M CdTe detector, with the detector translated slightly between measurements that have been corrected with (*a*) the factory detector FFC and (*b*) a recently calculated FFC. The measurements have different relative peak intensities when corrected with the factory FFC which cannot correspond to the sample structure, indicating that the detector flat-field response has changed over time. Intensities on the vertical axes are arbitrary, so no numbers are shown. Vertical magnification of the inset is 2×.

We observed that the translation of the detector changes the relative peak shapes and intensities of an amorphous scatterer without proper FFC. Simply translating the detector, taking a measurement and radially averaging provides 1D diffraction patterns that are not identical. Attempting to normalize these patterns based on their maximum intensity results in patterns with differing peak profiles. Correction of the detector data based on a measured FFC map removes this problem and results in 1D patterns which are in agreement regardless of the detector position (Fig. 7 ▸).

### 3.2. Changes in structural interpretation

**Figure 8:**
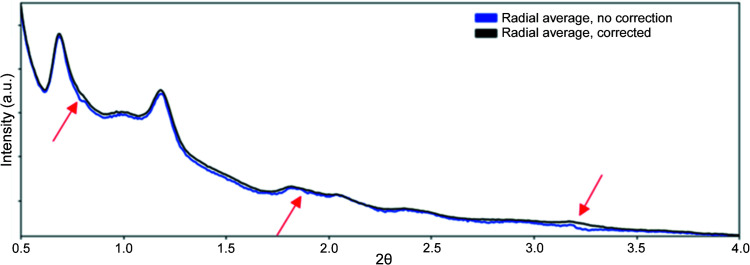
Radial averages of the scattering from carbon nanoparticles without FFC (blue) and with FFC (black), showing the presence of small peaks that are detector-response related, rather than sample related (major peaks labeled in red). Measurements were taken on 11-ID-C at 105.7 keV with the Pilatus 2M CdTe detector. Intensities on the vartical axes are arbitrary, so no numbers are shown.

For some materials, such as carbon nanoparticles, a diffraction pattern collected without FFC results in a 1D pattern with erroneous peaks that do not correspond to a real structural feature (Fig. 8 ▸).

**Figure 9:**
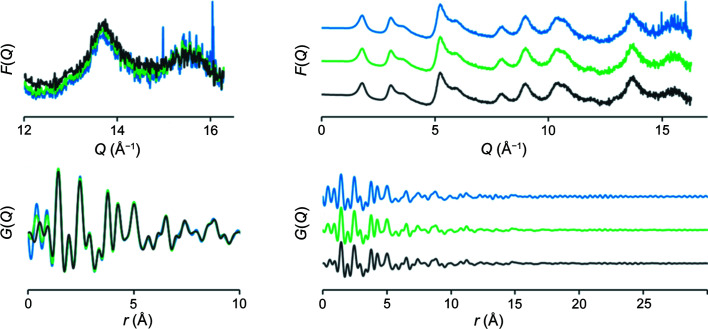
(*a*) *F*(*q*) functions calculated from Vulcan amorphous carbon with no correction (blue), hot pixel mask (green), hot pixel mask and FFC (black); and (*b*) the corresponding PDFs. A fit in *PDFgui* of the calculated PDFs with uncorrected (blue), masked (green) and FFC (black) results in fit residual values (*R*_w_) of 0.41, 0.36 and 0.28, respectively, with the best fit being to the FFC data.

When collecting data suitable for calculating PDFs from materials with low scattering intensity, low-crystallinity samples such as amorphous carbons or liquids, FFC significantly improves the signal-to-noise ratio and decreases artifacts in the data. Since most amorphous samples do not contain significant features at higher *Q* values in the measured *I*(*q*), any noise-related deviation of the signal in high-*Q* regions will be especially harmful to the measurements of these samples. Fig. 9 ▸(*a*) shows *F*(*Q*) data of amorphous carbon ‘Vulcan’. This is a high-surface-area, low-density material which contributes less than 10% of the total scattering signal, *I*(*q*), when measured in glass capillaries. In the uncorrected Vulcan measurement, the amplitude of variations across the uncorrected detector pixels is larger in magnitude than the data itself, resulting in significant noise in final PDF calculation. This appears as a ripple artifact in the final PDF, most easily visible at high *R* values. As a consequence of the ripple artifact, the fit to the uncorrected ‘Vulcan’ sample refined against the *P*6_3_ *mc* graphite structure within the *PDFgui* software (Farrow *et al.*, 2007 ▸) resulted in relatively high residuals (*R*_W_ = 0.41). In addition, the high-frequency noise at distances greater than 10 Å overpower low-amplitude features and may result in less accurate size estimations. Masking non-linearly responding pixels eliminates most of the obvious noise, lowering *R*_W_ by 5% (*R*_W_ = 0.36). A proper FFC further reduces both high-frequency and low-*r* noise bringing the *R*_W_ values down to 0.28. Fits done in *PDFgui* are shown in Fig. S5 of the supporting information.

### 3.3. Stability of detector corrections

**Figure 10:**
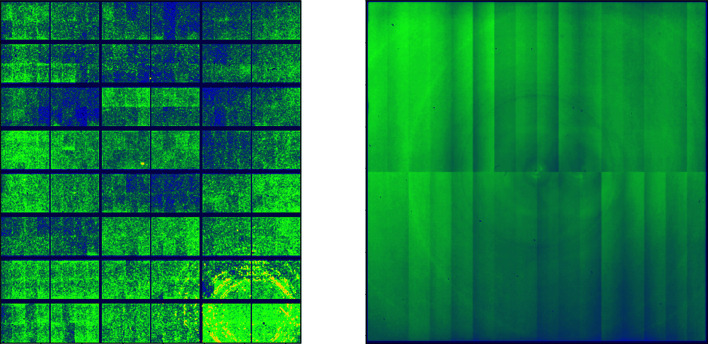
FFCs calculated for (*a*) Pilatus 2M CdTe and (*b*) Perkin Elmer XRD1621 detectors using data collected on beamline 11-ID-C at 105.7 keV. The energy threshold for the Pilatus 2M CdTe detector was set to 50 keV, and the FFC was found to match the map extrapolated from multiple flat-field calibrations automatically calculated by the detector computer.

Detector FFC maps were not found to vary significantly over the course of 12 h of continuous measurements with the Pilatus 2M detector. However, over the course of a month the FFC of a Pilatus 2M detector was found to change enough such that the radial average of measured material was slightly different (Section S2 of the supporting information). Corrected 2D scattering with a recent FFC map shows less of a ‘speckle pattern’ than the one corrected with an old FFC map, implying that the response of individual pixels is to drift slightly independent of the surrounding pixels [Fig. 2 ▸(*b*)]. FFC maps of the Pilatus 2M CdTe and Perkin Elmer XRD1621 detectors at 11-ID-C are shown in Fig. 10 ▸ and were calculated using measurements taken at 105.7 keV.

**Figure 11:**
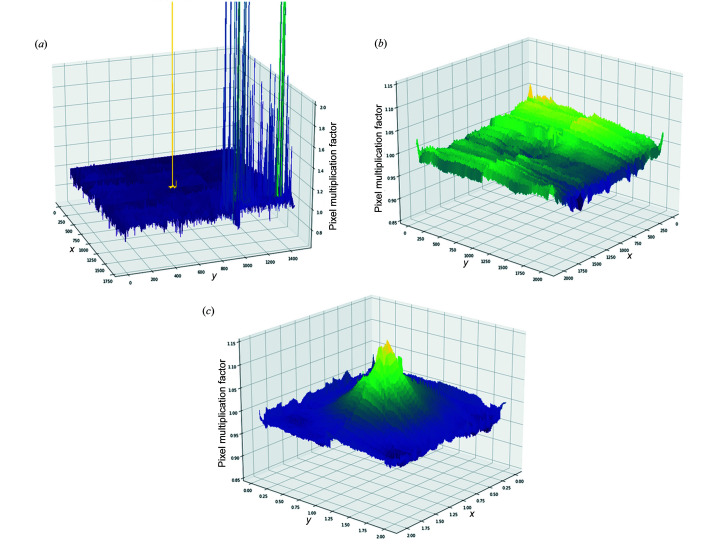
FFC maps displayed as height maps for (*a*) the Pilatus 2M CdTe detector at beamline 11-ID-C, (*b*) the Perkin Elmer XRD1621 at beamline 11-ID-C and (*c*) the Perkin Elmer XRD1621 at beamline 11-ID-B. Very large spikes in the Pilatus 2M CdTe detector are characteristic of radiation damage, with the largest spike in the center of the detector being the result of direct exposure to the X-ray beam. The large conical structure in the FFC map of the Perkin Elmer XRD1621 at 11-ID-B is the result of heavy radiation damage caused by use over many years and is characteristic of a detector that should be retired. The relatively flat FFC of the Perkin Elmer XRD1621 at 11-ID-C is characteristic of a normal-functioning detector.

Displaying calculated FFC for detectors as a height map is a quick way to visualize regions damaged by radiation (Fig. 11 ▸) where large spikes appear. A detector with an FFC that appears as a conically shaped height map is indicative of radiation damage across the detector, and is likely a sign that the detector should be retired. The Perkin Elmer XRD1621 detector at 11-ID-B with such an FFC was also found to provide inaccurate diffraction measurements in later tests.

## 4. Conclusions

The ability to quickly calculate an FFC using measurements taken easily at a beamline in several minutes provides a number of advantages over the usual FFC calibration. The quantum efficiency of a detector varies with photon energy, resulting in a response at each pixel specific to the X-ray energy used. By using measurements taken at the photon energy at which a beamline operates, there is no longer a need to take several flat-field measurements at different photon energies using X-ray fluorescence and computing the detector FFC to extrapolate to the actual photon energy used at the beamline. FFC maps obtained by this method were found to be equivalent to those calculated from multiple X-ray fluorescence flat-fields. By using measurements that are obtained quickly and directly at the beamline, it becomes easier to monitor any flat-field response changes in the detector resulting from radiation damage. Additionally, a slight drift in detector FFC maps was observed over time scales of several weeks, suggesting a need to frequently recalibrate detectors with a more recent FFC in order to provide accurate measurements. Experiments that rely on the measurement of subtle changes in a measured signal, such as the measurement of PDFs of liquids and amorphous materials, will benefit from a recently calibrated detector directly before the experiment.

## Supplementary Material

Supporting figures and equations. DOI: 10.1107/S1600577523001157/gy5043sup1.pdf

## Figures and Tables

**Table 1:** Measurement parameters for FFC calculation at various beamlines The scatterer used for the measurements was a stack of 1.5 mm-thick glass microscope slides.

Beamline	Energy (keV)	Scatterer used	Collection time per position (min)	Sample-to-detector distance (mm)	Detector
17-BM-B	27	4 slides	12	400	Varex 4343CT
	51	4 slides	12	1000	Varex 4343CT
11-ID-B	58.6	8 slides	3	1000	PE XRD1621†
	86.7	8 slides	5	1000	PE XRD1621
11-ID-C	105.7	10 slides	2.5	1000	PE XRD1621†
11-ID-C	105.7	10 slides	2.5	1000	Pilatus 2M CdTe

† The PE XRD1621 detectors used were two different detectors of the same model.
