# *In situ* X-ray area detector flat-field correction at an operating photon energy without flat illumination. Corrigendum

**DOI:** 10.1107/S1600577523004320

**Published:** 2023-05-26

**Authors:** James Weng, Wenqian Xu, Kamila M. Wiaderek, Olaf J. Borkiewicz, Jiahui Chen, Robert B. Von Dreele, Leighanne C. Gallington, Uta Ruett

**Affiliations:** a X-ray Science Division, Advanced Photon Source, Argonne National Laboratory, Lemont, IL 60439, USA; Paul Scherrer Institut, Switzerland

**Keywords:** flat-field correction, flat-field calibration, dectector drift

## Abstract

The name of an author in the article by Weng *et al.* (2023) [*J. Synchrotron Rad.* **30**, 546–554] is corrected.

## Abstract 2

Corrigendum to the article by Weng *et al.* (2023) [*J. Synchrotron Rad.* **30**, 546–554].

The name of the fifth author in the article by Weng *et al.* (2023 ▸) is incorrectly given as Jahui Chen. The correct spelling is Jiahui Chen, as given above.
