# Supplementary material for: In situ X-ray area detector flat-field correction at an operating photon energy without flat illumination
Source: J Synchrotron Radiat. 2023 Mar 10;30(Pt 3):546–54. doi: 10.1107/S1600577523001157 (PMC10161894; doi:10.1107/S1600577523001157)
Supplement: Supplementary file 1 [file s-30-00546-sup1.pdf]

JOURNAL OF
SYNCHROTRON
RADIATION

Volume 30 (2023)

Supporting information for article:

in situ X-ray area detector flat field correction at an operating photon energy without flat illumination

James Weng, Wenqian Xu, Kamila M. Wiaderek, Olaf J. Borkiewicz, Jahui Chen, Robert B. Von Dreele, Leighanne C. Gallington and Uta Ruett

S1. Example measurements

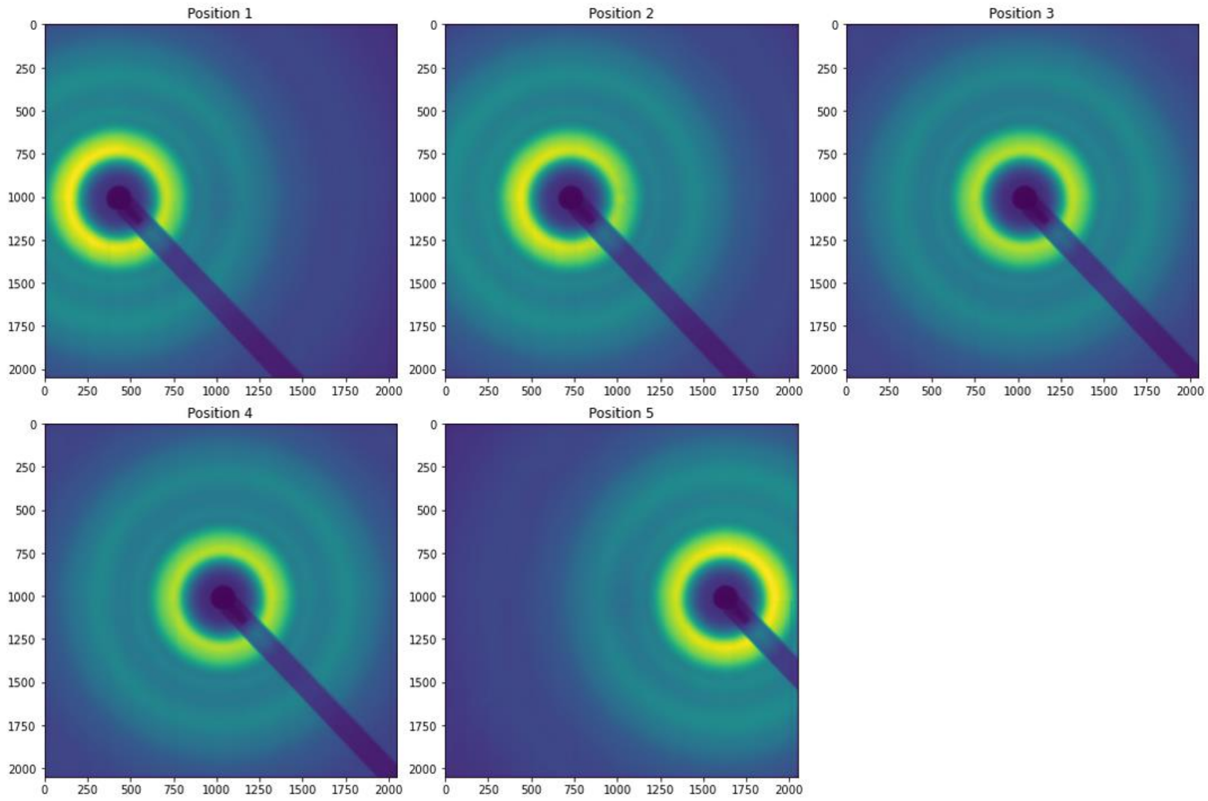

Figure S1 2D Diffraction measurements with an identical sample and the detector translated to five different positions used to calculate a flat field correction. The translation distance of the detector between the measurements is not critical, so long as the occluded beam stop regions do not overlap between the measurements.

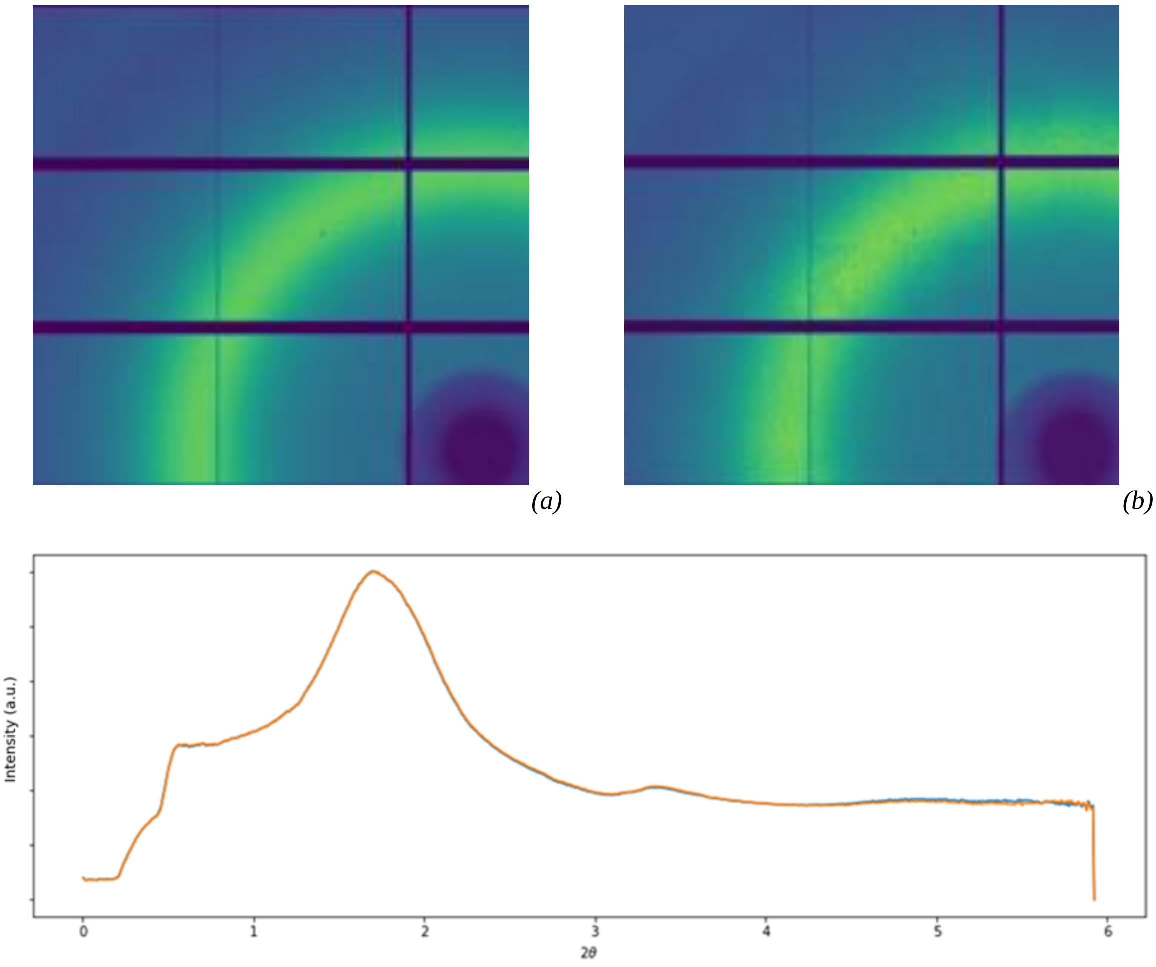

(a) *(b)*

(c)

Figure S2 Measured 2D scattering corrected using a recently collected flat field correction (a) and a month old flat field correction (b) along with the corresponding radial averages (c). Radial average from the recent flat field correction is shown in orange and the old flat field correction is shown in blue. Corrected 2D scattering with a recent flat field correction shows less of a “speckle pattern” than the one corrected with an old flat field correction, implying that the response of individual pixels drift slightly independent of the surrounding pixels. Vertical axis is arbitrary so no values are shown.

S2. S2 Detector tilt on pixel intensity

When the detector is perfectly perpendicular to the incident X-ray, the scattered X-ray travel the same length of path to reach all the pixels at the same 2θ angles (Fig. S3a). In a realistic condition where a tilt angle is present, shown as α in Fig. S3b, pixels of the same 2θ angles have different distances to the scattering point on the sample (S). The closest and farthest pixels are the two along the tilt direction, shown as B and D. The photon density at point B and D should be inversely related to the square of their pixel to sample distances,

$$\rho_B/\rho_D = |SD|^2/|SB|^2$$

where ρ_B is the photon density at point B, $|SD|$ is the distance between S and D. The lengths of relevant line segments in Fig. S3b can be expressed using θ and α.

$$|AB| = |AO|\sin\alpha/\sin(\pi/2 + 2\theta - \alpha) \approx |AO|\,\alpha/\cos 2\theta$$

$$|CD| = |CO|\sin\alpha/\sin(\pi/2 - 2\theta - \alpha) \approx |CO|\,\alpha/\cos 2\theta$$

$$|AO| = |CO|$$

$$|AS| = |CS| = |AO|/\sin 2\theta$$

$$\rho_B/\rho_D = |SD|^2/|SB|^2 = \left(\frac{|CS| + |CD|}{|AS| - |AB|}\right)^2 = \left(\frac{1 + \alpha\tan 2\theta}{1 - \alpha\tan 2\theta}\right)^2 \approx 1 + 4\alpha\tan 2\theta$$

Another effect of tilting is the apparent pixel size at B is different from that at D,

$$S_B = S\cos(2\theta - \alpha)$$

$$S_D = S\cos(2\theta + \alpha)$$

where S is the actual size of the pixel, S_B is the apparent size at point B, which is the area of the pixel projected to the plane normal to the scattered beam direction. The ratio of the two apparent sizes,

$$S_B/S_D \approx \frac{\cos 2\theta + \alpha\sin 2\theta}{\cos 2\theta - \alpha\sin 2\theta} \approx 1 + 2\alpha\tan 2\theta$$

We can then derive the ratio of intensity measured by pixels at B and D,

$$I_B/I_D = \frac{\rho_B S_B}{\rho_D S_D} \approx 1 + 6\alpha\tan 2\theta$$

In the study of the Pilatus detector, the tilt was calibrated as 0.97°, the max 2θ was 6°, so the term $6\alpha\tan 2\theta$ = 0.011. And this 1.1% deviation is the calculated maximum deviation along the tilt direction between the two pixels at opposite edges. This is much less than the ~20% variation in detector response among the pixels found in the Pilatus detector.

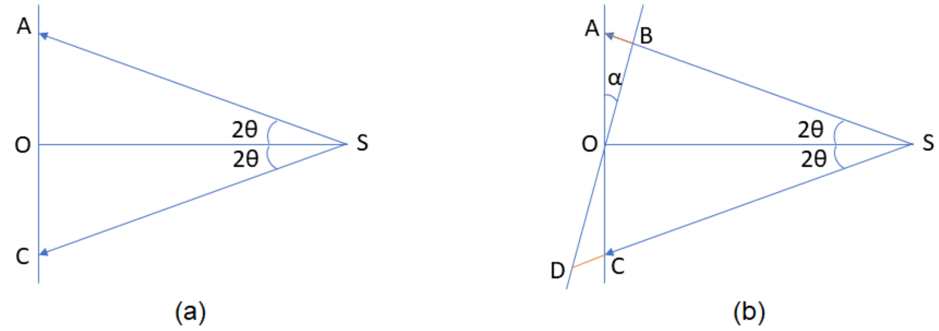

Figure S3 Diagrams of detector without tilt (a) and with a tilt angle of α (b). The tilt leads to different lengths of X-ray travel path from sample to pixel as well as different apparent pixel sizes.

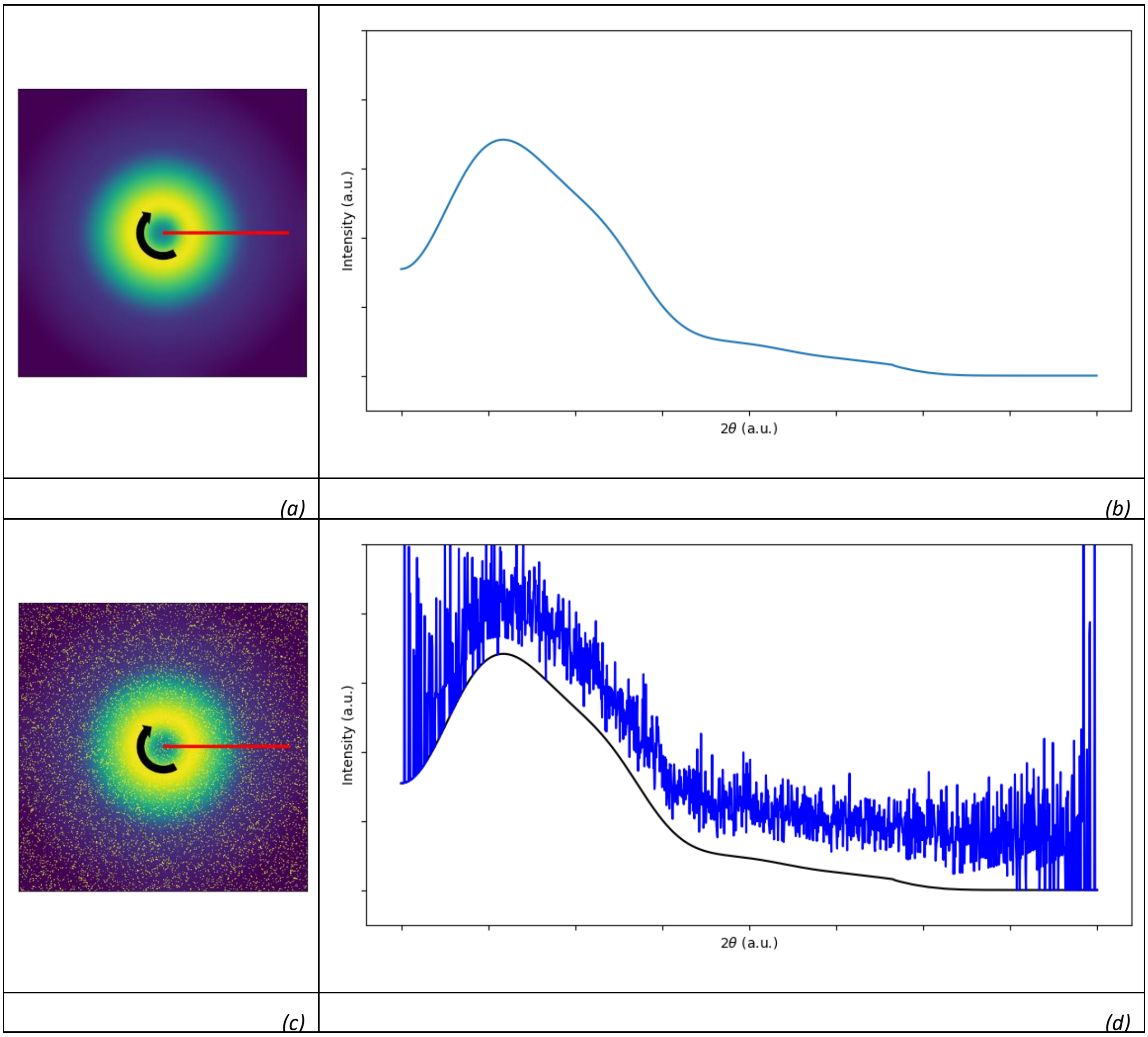

Figure S4 A simulated 2D diffraction pattern of an amorphous scatterer with no measurement noise (a) and its corresponding radial average (b). The radial average is taken around the rotation axis denoted by the black arrow. The addition of outliers to the 2D pattern (c) significantly corrupts the radial average (d) if a mean is used (blue) while the median provides the expected radial average (black).

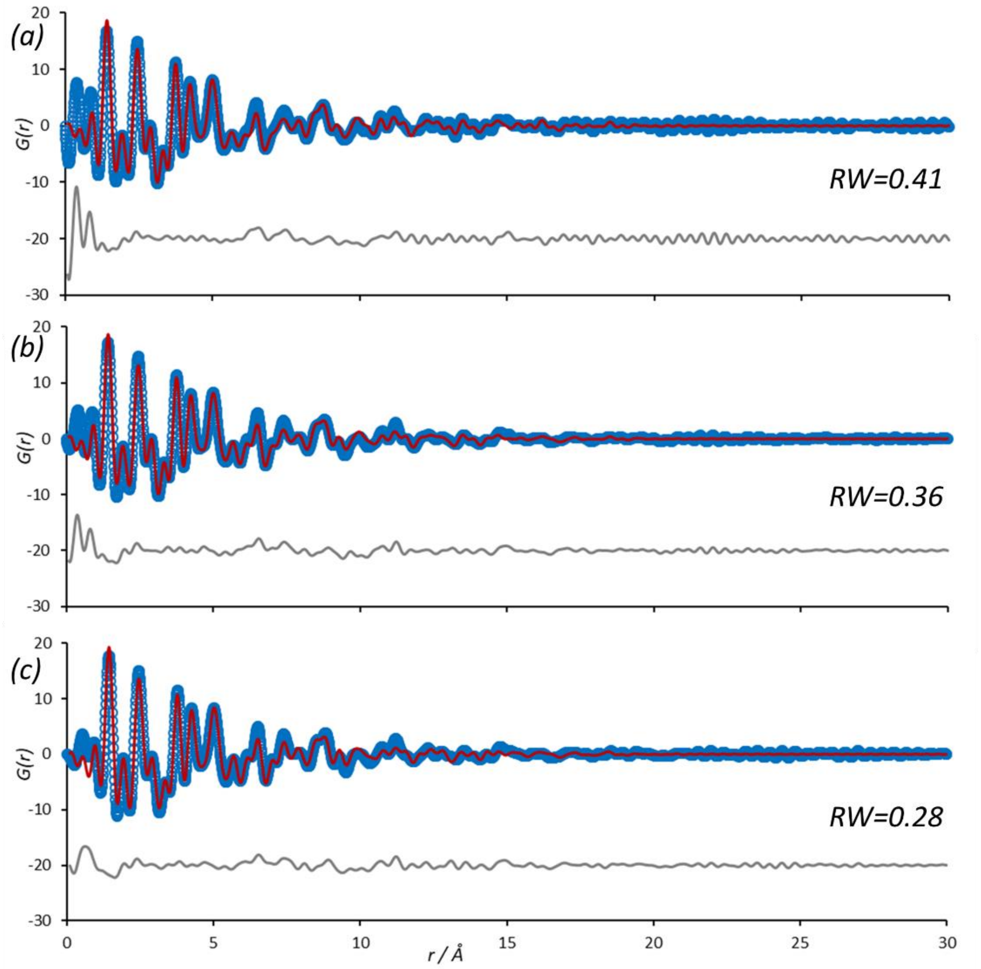

Figure S5 Fits of calculated PDFs from the amorphous carbon material “Vulcan” calculated from data without an applied flat field correction (a), a pixel mask removing heavily radiation damaged pixels (b), and flat field corrected data (c). PDF calculated from the uncorrected data shows more ripple artifacts, most easily visible above r = 15 and below r = 1. The fit residuals for the masked and flat field corrected data are lower than the uncorrected data, with the flat field corrected data resulting in the lowest fit residual.
